# A barcode of organellar genome polymorphisms identifies the geographic origin of *Plasmodium falciparum* strains

**DOI:** 10.1038/ncomms5052

**Published:** 2014-06-13

**Authors:** Mark D. Preston, Susana Campino, Samuel A. Assefa, Diego F. Echeverry, Harold Ocholla, Alfred Amambua-Ngwa, Lindsay B. Stewart, David J. Conway, Steffen Borrmann, Pascal Michon, Issaka Zongo, Jean-Bosco Ouédraogo, Abdoulaye A. Djimde, Ogobara K. Doumbo, Francois Nosten, Arnab Pain, Teun Bousema, Chris J. Drakeley, Rick M. Fairhurst, Colin J. Sutherland, Cally Roper, Taane G. Clark

**Affiliations:** 1 Immunology and Infection Department, London School of Hygiene and Tropical Medicine, London WC1E 7HT, UK; 2 Malaria Programme, Wellcome Trust Sanger Institute, Hinxton CB10 1SA, UK; 3 Department of Entomology, Purdue University, West Lafayette, Indiana 47907, USA; 4 International Center for Medical Research and Training, Carerra 125, Cali, Colombia; 5 College of Medicine, University of Malawi, Blantyre 3, Malawi; 6 Malawi-Liverpool-Wellcome Trust Clinical Research Programme, Blantyre 30096 BT3, Malawi; 7 Malaria Capacity Development Consortium, Liverpool School of Tropical Medicine, Liverpool L3 5QA, UK; 8 Medical Research Council Laboratories (UK), Fajara PO Box 273, The Gambia; 9 KEMRI-Wellcome Trust Research Programme, Kilifi 80108, Kenya; 10 Department of Parasitology, Institute of Tropical Medicine, University of Tübingen, Tübingen 72074, Germany; 11 Department of Rural Health, Faculty of Health Sciences, Divine Word University, Madang PO Box 483, Papua New Guinea; 12 Institut de Recherche en Sciences de la Sant, Bobo-Dioulasso BP 545, Burkina Faso; 13 Malaria Research and Training Centre, Faculty of Medicine, Pharmacy and Dentistry, University of Bamako, Bamako BP1805, Mali; 14 Centre for Tropical Medicine, Nuffield Department of Medicine, University of Oxford, Oxford OX3 9DS, UK; 15 Shoklo Malaria Research Unit, Mahidol-Oxford Tropical Medicine Research Unit, Faculty of Tropical Medicine, Mahidol University, Mae Sot 63110, Thailand; 16 Pathogen Genomics Laboratory, King Abdullah University of Science and Technology, Thuwal 23955-6900, Kingdom of Saudi Arabia; 17 Malaria Pathogenesis and Human Immunity Unit, Laboratory of Malaria and Vector Research, National Institute of Allergy and Infectious Diseases, National Institutes of Health, Bethesda, Maryland 20892, USA

## Abstract

Malaria is a major public health problem that is actively being addressed in a global eradication campaign. Increased population mobility through international air travel has elevated the risk of re-introducing parasites to elimination areas and dispersing drug-resistant parasites to new regions. A simple genetic marker that quickly and accurately identifies the geographic origin of infections would be a valuable public health tool for locating the source of imported outbreaks. Here we analyse the mitochondrion and apicoplast genomes of 711 *Plasmodium falciparum* isolates from 14 countries, and find evidence that they are non-recombining and co-inherited. The high degree of linkage produces a panel of relatively few single-nucleotide polymorphisms (SNPs) that is geographically informative. We design a 23-SNP barcode that is highly predictive (~92%) and easily adapted to aid case management in the field and survey parasite migration worldwide.

## Abstract (web summary)

Tracing the source of malarial infections is an important step towards monitoring and controlling the disease. Here, Preston *et al*. analyse sequence data from 711 isolates and design a genetic barcode based on combined mitochondrial and apicoplast genomes that is able to distinguish between malaria parasites isolated from different geographical regions.

## Graphical abstract

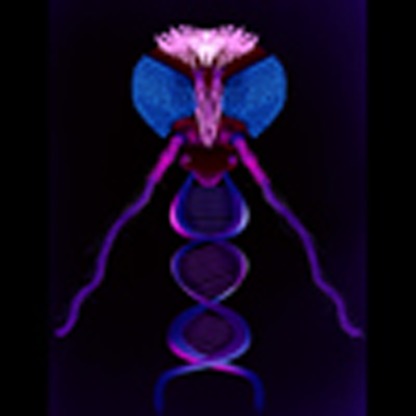

Malaria threatens nearly half the world’s population, and the deadliest form caused by *Plasmodium falciparum* remains a leading cause of childhood mortality worldwide^1^. As countries move closer to elimination and parasites develop tolerance of artemisinins^2^,^3^, understanding the inter-connectedness of parasite populations and tracing the source of imported infections have become top priorities. Genetic markers have proved extremely valuable in the eradication of other diseases (for example, polio^4^). Analysis of nuclear genome variation in *P. falciparum* (14 chromosomes, 23 Mbp, 19.1% GC content) has been used to identify candidate artemisinin-resistant loci^3^, and can be exploited to map the dispersion of parasites worldwide and trace the migration of drug-resistant parasites into new areas. Thus, a universal *P. falciparum* genotyping tool able to interrogate geographically restricted single-nucleotide polymorphisms (SNPs) would be of great value. Current barcoding approaches^5^ based on nuclear SNPs are constrained by a lack of geographic specificity and frequent recombination, which disrupts multi-locus SNP associations in each generation. To overcome these limitations, we explored the usefulness of the extra-nuclear genomes of the mitochondrion and apicoplast organelles. We postulated that strict maternal inheritance might exclude recombination and so create a barcode that is stable and geographically informative over time.

The mitochondrion genome (*mt*) of *P. falciparum* is a 6-kb concatenated linear sequence, is transmitted in female gametocytes and does not recombine among lineages^6^,^7^,^8^; thus, sequence polymorphism in *mt* is attractive as a potential barcoding tool. Analysis of global sequence variation in *mt* has revealed geographic differentiation^9^,^10^,^11^, but the limited numbers of SNPs restrict its capacity to resolve fine-scale population differentiation. Apicoplasts are relict non-photosynthetic plastids found in most protozoan parasites belonging to the phylum Apicomplexa, including all *Plasmodium* species, and show phylogenetic homology to the chloroplasts of plants and red algae^12^,^13^. Although the apicoplast has lost any ancestral photosynthetic ability, it retains a genome encoding lifecycle-specific, essential metabolic and biosynthetic pathways that generate isoprenoids, fatty acids and haem^13^. As these are distinct from homologous human pathways, the apicoplast is an enticing target for antimalarial drugs^13^,^14^,^15^,^16^,^17^. The apicoplast genome (*apico*) is a 35-kb circular sequence^6^ and is also maternally inherited. Although polymorphism in *apico* (29.4 kb annotated core, 30 genes, 13.1% GC content) has not been well characterized, it is potentially greater than that in *mt* (6 kb, 3 genes, 31.6% GC content) owing to its larger size. To develop a robust *mt/apico* barcode and improve our understanding of *apico* evolution, a definitive analysis of *apico* SNP variation in multiple *P. falciparum* populations is needed to determine the extent of global diversity and existence of recombination.

Although there is good evidence that chloroplasts and mitochondria are co-inherited in plants^18^, this is not a hard-and-fast rule in other organisms^19^. Evidence from the laboratory indicates that *mt* and *apico* are co-transmitted during *P. falciparum* gametocytogenesis^7^, but evidence from the field is lacking. Here, using sequence data from 711 parasite isolates in 14 countries across four continents, we catalogue 151 *mt* SNPs and 488 *apico* SNPs and use them to investigate organelle DNA co-inheritance and geographic differentiation at the population level. We find high linkage disequilibrium (LD) between *mt* and *apico* SNPs within populations, providing strong evidence that the organelles are indeed co-transmitted and non-recombining. This finding represents a breakthrough in the genetic barcoding of *P. falciparum*, as it reveals novel extended haplotypes specific for different geographic settings. Using SNP variation of the combined organelle genome (*mt/apico*) in an iterative haplotype-based classification analysis, we construct a 23-SNP barcode that identifies the region of sample origin with 92% predictive accuracy.

## Results

**Figure 1:**
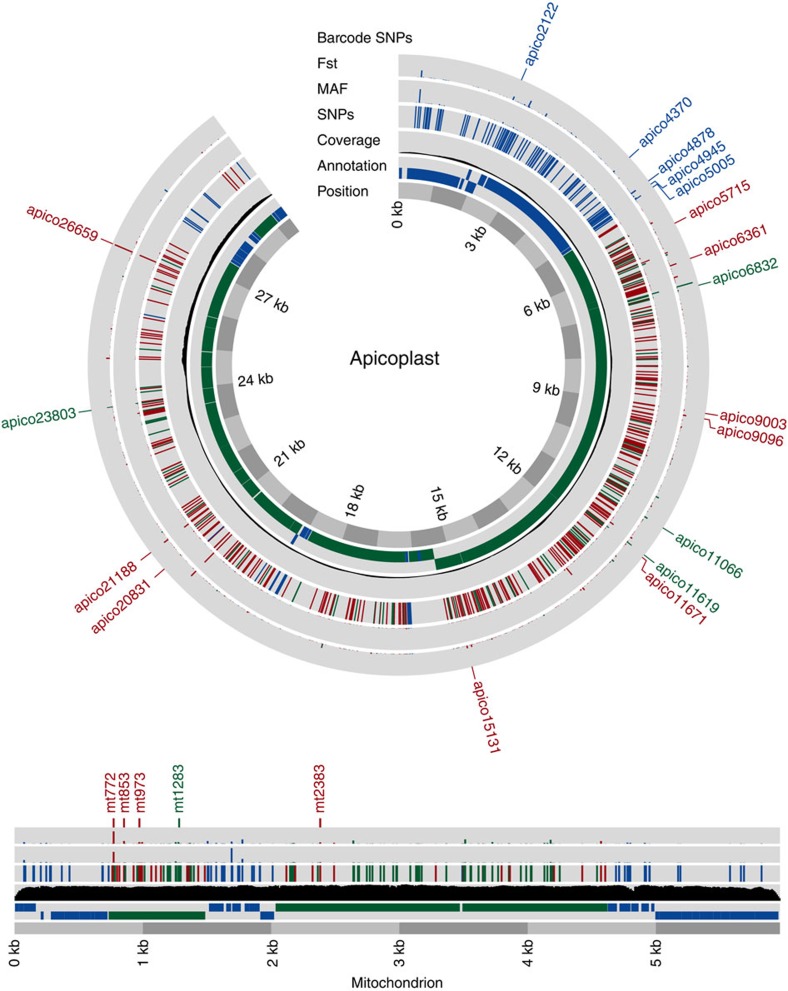
*Plasmodium falciparum* mitochondrion and apicoplast genomes. The nucleotide sequence landscape of the densely packed *P. falciparum* mitochondrion (*mt*) and apicoplast (*apico*) genomes. Protein-coding (green) and non-translated RNA (blue) regions in the ‘annotation’ ring are transcribed from either strand (inner, negative strand; outer, positive strand). The 20-fold difference in coverage between the genomes is visible (see also Supplementary Fig. 1). All mutations within *mt* (151 SNPs, 5,967-bp linear) and *apico* core (488 SNPs, 29,430-bp circular, excluding an inverted repeat) are shown relative to the *P. falciparum* 3D7 (version 3.0) reference genome coordinates. SNPs are densely packed throughout, with more non-synonymous (NS) protein-coding changes (red) in *apico* than in *mt*. Synonymous, intronic, intra-genic (green) and RNA changes (blue) are also marked. The minor allele frequency (MAF), *Fst* and barcode SNPs are marked in the outer three rings and are colour coded in the same way (the full catalogue is available online). The 23 barcoding SNPs (5 *mt*, 4 NS; 18 *apico*, 9 NS) are marked in the outer ring.

To the 3D7 reference genome we aligned high-quality raw sequence data from 711 *P. falciparum* samples in five geographic regions: West Africa (WAF: Burkina Faso, Gambia, Ghana and Mali, *N*=401), East Africa (EAF: Kenya, Malawi and Uganda, *N*=98), Southeast Asia (SEA: Cambodia, Thailand and Vietnam, *N*=164), Oceania (OCE: Papua New Guinea, *N*=25) and South America (SAM: Colombia and Peru, *N*=23). The sequence coverages of *mt* (~1,000-fold) and *apico* (~100-fold) are ~22-fold and ~two-fold greater than the nuclear genome, respectively (Fig. 1; Supplementary Fig. 1). These fold differences in coverage are consistent with known organelle copy numbers in single *P. falciparum* parasites^20^,^21^. Using all sample alignments, we identified 151 high-quality SNPs in *mt* (25.3 SNPs per kilobase, 65.6% in coding regions) and 488 in *apico* (16.6 per kilobase, 77.5% in coding regions) (Supplementary Table 1). Of the 151 SNPs, only 20 (13.2%) were identified previously^9^,^10^. Across all samples, 65.4% (418/639) of SNPs were singletons, 92.5% (591/639) were rare (minor allele frequency, MAF <1%), 7.5% (48/639) had a MAF >1% and 2.3% (15/639, 3 *mt* and 12 *apico*) were common (MAF >5%) (Supplementary Table 1). Multi-allelic SNPs were identified in both genomes (*mt* 4.0%, *apico* 5.1%); 29 were tri-allelic and two were quad-allelic (Supplementary Table 1). Of the multi-allelic SNPs, only the quad-allelic locus *mt1692* described previously^10^ has a combined MAF >5%.

Geographic patterns of diversity were investigated by linear discriminant analysis of the combined *mt* and *apico* SNP data, which revealed clustering by geographic origin of samples (Supplementary Fig. 2). To determine the most significant drivers of population differentiation, we analysed only non-rare SNPs. We calculated population differentiation statistic *Fst* to identify SNPs with inter-regional allele frequency differences, which range from 0 to 1 with higher values signifying greater differentiation^22^ (Fig. 1). We found substantially lower population differentiation between countries in the same region (mean 28.5 SNPs per region with *Fst* >0.05) than between the five regions (mean 58 SNPs with *Fst* >0.05). Forty-nine SNPs have MAF >1% in at least one region (Supplementary Table 2), 17 (34.7%) of which have *Fst* >0.1 (Supplementary Fig. 3). Of these 17 (4 *mt* and 13 *apico*), 14 are located in genes with 8 non-synonymous (NS) changes (Supplementary Fig. 3; Supplementary Table 2). The two SNP loci with highest *Fst* (~0.76), *mt772* (*cox3*) and *apico6762* (*orf101*), are in perfect LD (*r*^2^=1) and differentiate SEA from other regions (MAFs: overall 16.5%, SAM 0%, WAF 0%, EAF 0%, SEA 69.0% and OCE 20.8%). A third SNP with high *Fst* (~0.88), *apico*26659 (*rpl23*), differentiates Africa (WAF and EAF) and SAM from other regions (Supplementary Fig. 3; Supplementary Table 2).

To assess the extent of recombination between SNPs within and between *mt* and *apico*, we carried out intra- and inter-region analyses of LD. Using non-rare biallelic markers, there was near-perfect LD between the combined *mt* and *apico* SNPs, within and across geographic regions. This is strong evidence that there is no recombination within or between organelles (mean pairwise *D′*=0.998 for all regions combined, Supplementary Fig. 4), the latter implying potential co-transmission. To investigate this possibility, we used 146 haplotypes (the observed combinations of SNPs in individual parasite isolates) of *mt* and 271 haplotypes of *apico*, respectively (Supplementary Table 1). By comparing the joint *mt*/*apico* haplotype frequencies (Supplementary Table 3), we found that the dependence between *mt* and *apico* was highly significant (*χ*^2^=64,921, d.f.=39,566, *P*<10^−16^), providing strong evidence of co-inheritance of the two organelles. This genetic evidence confirms the experimentally observed and theoretically predicted processes involved in gametocytogenesis^6^,^7^.

The geographical pattern of *mt* haplotypes was previously interpreted to reflect radiation of *P. falciparum* out of Africa into SEA and SAM^10^. Consistent with this interpretation, our analysis of 151 *mt* SNPs identifies a common haplotype in 30.0% (213/711) of samples, which is represented in four of the five regions: SAM 30.4%, WAF 37.2%, EAF 49.0%, SEA 0% and OCE 36% (Supplementary Fig. 5). Since this compromises geographical assignment, *mt* haplotypes alone cannot identify the geographic origin of parasite strains. The addition of 488 *apico* SNPs to generate 290 distinct compound (*mt*/*apico*) haplotypes greatly increases the geographic resolution of samples (Supplementary Fig. 5). Nearly all (282/290, 97.2%) compound haplotypes are observed in one region only, and 66.8% of all parasite isolates have a haplotype unique to their region of origin. Six of the eight *mt*/*apico* haplotypes observed in multiple regions are most common in Africa (WAF and EAF), consistent with an African origin for this parasite species.

**Figure 2:**
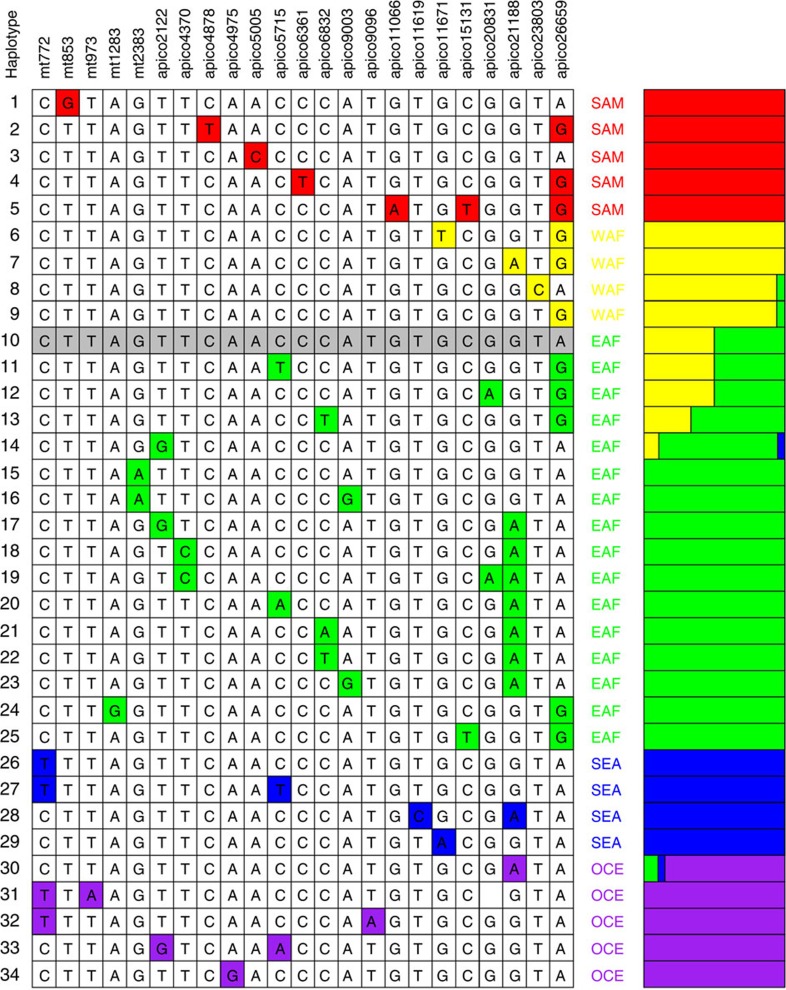
SNP barcode across *P. falciparum* mitochondrion and apicoplast genomes. The 23 SNP loci form 34 distinct haplotypes that help identify a parasite’s geographical origin: South America, SAM; West Africa, WAF; East Africa, EAF; Southeast Asia, SEA; and Oceania, OCE. Most (76.5%, 26/34) haplotypes are unique to a single region. Haplotype 10 corresponds to the 3D7 reference strain, and its mitochondrion (*mt)* core haplotype is observed in all five regions. Two haplotypes (14 and 30) are seen in three regions. The overall accuracy is 92.1% (655/711; SAM 100%, WAF 94.5%, EAF 68.4%, SEA 98.8% and OCE 96.0%).

After discovering the existence of regional differentiation, we sought to identify a minimal set of barcoding SNPs diagnostic for the compound *mt*/*apico* haplotypes. Using the 221 SNP loci with non-singleton alleles, we applied an iterative haplotype search algorithm that maximized predictive accuracy, while accounting for regional sample size differences and avoiding over-fitting. The minimal barcode comprises 23 SNPs (5 *mt*, 18 *apico*, MAF >1% in a single region, 3 tri-allelic), within 18 protein-coding genes (13 NS), four non-translated RNA segments and one inter-genic region (Supplementary Table 2; Fig. 1). The 23 SNPs form only 34 distinct haplotypes (Fig. 2), 26 of which are unique to one region. The core 3D7 haplotype 10 occurs in 14 African isolates (2.8%, 7 WAF and 7 EAF). Haplotypes 14 and 30 occur in three regions and deviate from the core by single mutations.

The overall predictive accuracy of the minimal barcode is 92.1% (655/711, Supplementary Table 4), compared with 95.1% (676/711) using all 639 *mt* and *apico* SNPs, and 82.1% using 24 nuclear SNPs^5^ (Supplementary Fig. 5). Across all regions except EAF, the predictive accuracy using the barcode is at least 94%. Almost half the discrepancies (24/56) arise from EAF samples being assigned to WAF. The high diversity in EAF samples leads to poor identification using the full and barcoding sets of SNPs, highlighting the need for further characterization of sample genomes from this region. The 23-SNP barcode was validated on sequence data from 81 *P. falciparum* samples not used in its construction, including five laboratory-adapted clones (3D7, HB3, 7G8, DD2 and GB4 (ref. 23)^23^), eight samples from travellers returning to London from EAF or WAF^24^, 154 samples from Africa (Senegal, *N*=12 (ref. 25)^25^; Ghana, *N*=16; Guinea, *N*=106 (ref. 26)^26^; Malawi, *N*=20 (ref. 27)^27^) and 20 samples from SEA^28^. The geographic origins of 93.0% (174/187) of isolates were correctly assigned; the origins of eight Malawian (EAF) and five Guinean (WAF) parasites were unassigned as their haplotypes are found in both East and West Africa (haplotypes 8–14, see Fig. 2).

## Discussion

Worldwide genetic variation in *P. falciparum* reflects population history, demography and geographic distance; however, recombination disrupts signals of differentiation in the nuclear genome, and since organelle sequence is non-recombining it can be uniquely informative when tracing patterns of dispersal. Mitochondrial and chloroplast sequences are commonly used in DNA barcodes for animals and plants^29^ and have been used to explore the origins of humans^30^ and wine grapevines^31^. Using genetic variation in the multi-copy *mt* and *apico* genomes, we have established a 23-SNP barcode that is geographically informative and robust to the effects of recombination. Rapid sequencing and genotyping technologies can be applied to small amounts of relatively low-grade parasite material, such as that sourced from finger-prick bloodspots. Exploiting uniqueness in the sequences surrounding the informative SNPs supports highly specific identification. The application of this tool has the potential to improve the management of imported cases and reduce the risk of local epidemics resulting from further transmission. Hence it will be a valuable tool for local agencies in programmes of malaria elimination and resistance containment.

The geographic differentiation seen in organelle genomes may also be subject to evolutionary forces in addition to genetic drift and migration. The presence of a core haplotype is consistent with *P. falciparum* radiation from Africa in the recent past, while sequence analysis using Tajima’s *D* metric^32^ supports population expansion in Africa and Asia, and possibly Oceania, and suggests a neutrally mutating population in South America (Supplementary Fig. 6)—all consistent with previous studies of mitochondrial genome diversity^10^. We explored the possibility that selective forces are also influential. Drug pressure, for example, is exerted regionally through sequential roll-outs of new antimalarial treatments in response to emerging drug resistance. The resulting selective sweeps identified in the nuclear genome have regional dispersal patterns^25^,^33^,^34^,^35^. In the mitochondrial genome, mutation in codon 268 of *cytb* occurred *in vitro* in the presence of atovaquone-proguanil selection^11^. However, we previously observed no naturally occurring polymorphisms in codons 133, 268 or 280 of this gene^25^. Since the mitochondrion is a putative target of the antimalarial action of artemisinins^33^, we looked for association between non-rare *mt/apico* SNPs and putative artemisinin-resistant loci (chromosome 13 region^3^ and *UBP1* (ref. 36)) but found weak correlation (mean *r*^2^=0.00257, maximum *r*^2^=0.515). We also considered nuclear SNPs known to be associated with resistance to chloroquine (*crt*, *mdr1*, mean *r*^2^=0.00454, maximum *r*^2^=0.621) and antifolates (*dhfr*, *dhps*, mean *r*^2^=0.00837, maximum *r*^2^=0.371), but again found only weak correlation.

A striking observation is the high proportion of NS changes among coding SNPs in *apico* (77.8%) compared with *mt* (31.3%) and the nuclear (61.8%) genome^27^, which may suggest they are subject to different selective pressures. While all *mt* genes have low NS to S ratios, indicative of purifying selection and a conserved functional role, *apico* genes generally have high NS to S ratios indicative of divergence and directional selection^37^. Drugs may exert selection; the highest NS ratios were in *rp8*, *rps7* and *tufA* (Supplementary Table 5), the latter encoding a target of the antibiotic thiostrepton and its derivatives^38^. A more prosaic explanation is nucleotide bias through the unusual *apico* DNA replication machinery^39^. To explore this further, we compared NS to S ratios among *apico*-encoded proteins and 545 nuclear-encoded *apicoplast* proteins^40^. The high rate appears to be confined to those apicoplast proteins encoded in *apico* itself (77.8% NS) rather than the nuclear genome (60.6% NS), thus supporting the DNA replication hypothesis. A similar analysis of *mt*-encoded proteins and 381 nuclear-encoded mitochondrial proteins^41^ found NS rates of 55.6% in the nuclear genome and 31.3% in *mt*. This points to a conservation mechanism that is intrinsic to the mitochondrial sequence. It is also significant that the absence of recombination introduces a constraint on the selective removal of slightly deleterious mutations^42^, and it is possible that mutations accumulate in sequences linked to genes under strong directional selection. However, multi-copy states of *mt* and *apico* within individual parasites may allow deleterious copies to be jettisoned by intracellular selection.

The apicoplast shares evolutionary similarities with the chloroplasts of photosynthetic eukaryotes and the prokaryotic progenitors of all plastids, and is vital to the survival of *Plasmodium* species^13^,^17^. The organelle thus encodes functions absent from vertebrate hosts and presents an enticing target for antimalarial drugs^14^,^15^,^16^,^17^, including novel applications of known antibiotics and herbicides. By combining these insights with reverse-genetic approaches, it may be possible to identify key proteins and metabolic pathways as new candidate drug targets^15^ and to anticipate their effectiveness in geographically distinct parasite populations.

An ability to determine the geographic origin of *P. falciparum* isolates potentially has enormous practical utility in containing drug resistance and eliminating malaria. One potential limitation of the *mt/apico* barcode in its current form is the lack of representation of the Indian sub-continent, Central America, southern Africa and the Caribbean, owing to the scarcity of sequence data from these regions. In addition, there is a need to sample more intensively from EAF, a region of high genetic diversity, high migration and poor predictive ability. Once these data gaps are filled, the barcode can be re-calibrated to maximize its accuracy in assigning sample origin. The 23 SNPs can be modified in light of new sequence information to improve barcode specificity, especially for discriminating malaria importation from one or two known regions, in which case a minimal set can be applied. Adding genomic data from *P. vivax* and *P. knowlesi* should help broaden the scope of the barcode for pan-*Plasmodium* applications. Incorporating antimalarial drug-resistant loci^3^ will further enhance the usefulness of the barcode as an important tool for malaria control and elimination activities worldwide.

The demonstration that *mt and apico* sequences are non-recombining creates a new genotyping tool that is robust to the diluting effects of recombination. Global movement of parasites threatens elimination and treatment efficacy. By mapping global patterns of organellar genome polymorphism, we will gain new insights into the extent to which *P. falciparum* populations worldwide are inter-connected by international malaria migration.

## Methods

### Sequence data alignment and variant detection

Raw deep-sequence data (minimum read length 54 base pairs (bp)) were available from *P. falciparum* isolates sourced from Burkina Faso and Mali^27^,^28^,^43^,^44^,^45^, Ghana^43^, Gambia^27^,^43^,^46^, Guinea^26^, Kenya^28^,^36^, Malawi^27^, Thailand and Cambodia^27^,^28^,^43^,^45^, Colombia^47^ and Vietnam^43^, as well as laboratory-adapted clones (DD2, HB3, 7G8 and GB4 (ref. 23)^23^) (also see ref. 27). *mt* sequence data for 101 samples (SAM 26, WAF 20, EAF 8, SEA 30, OCE 11 and other 6) were also available^10^.

All sequences were mapped uniquely onto the 3D7 reference genome (14 chromosomes, 23 Mb; mitochondrion, 6 kb; apicoplast core, 30 kb; version 3.0) using *smalt* alignment software (www.sanger.ac.uk/resources/software/smalt) with default settings within an established pipeline^24^,^27^. The resulting alignments enabled the identification of high-quality (Q30) SNPs and small insertions/deletions (indels) using SAMtools and BCF/VCF tools (samtools.sourceforge.net). Genotypes were called using coverage as described^24^,^27^, where a minimum of 10-reads support was required to call an allele.

### Population genetics and statistical analysis

A linear discriminant analysis was performed to cluster parasite isolates on the basis of genetic information, specifically using pairwise identity by state based on SNP allele differences. SNPs identified in the nuclear genome (~600 K SNPs, http://pathogenseq.lshtm.ac.uk/plasmoview^27^) were used in a principal component analysis to identify potential geographical outliers. Analyses of allele frequency distributions were performed using within-population Tajima’s *D* indices^32^ and between-population *Fst*^22^. Negative Tajima’s *D* values signify an excess of low-frequency polymorphisms relative to expectation, indicating population size expansion (for example, after a bottleneck or a selective sweep) and/or purifying selection. Positive Tajima’s *D* values signify low levels of low- and high-frequency polymorphisms, indicating a decrease in population size and/or balancing selection. *Fst* metric values range from 0 (equivalent allele frequencies across populations) to 1 (complete differentiation for at least one population). The *Ka/Ks* ratio was calculated as an indicator of selective pressure acting on a protein-coding gene (Supplementary Table 5). It is the ratio of the number of NS substitutions per NS site (*Ka*) to the number of synonymous (S) substitutions per S site (*Ks*)^48^. Increasing values of *Ka/Ks* from 1 imply positive selection, while values decreasing from 1 imply purifying selection. LD was assessed using pairwise *D'* and *r*^*2*^ methods^49^. The barcode was constructed using an iterative SNP algorithm that considers the classification of regions using haplotypes, attempting to maximize predictive accuracy (weighted or unweighted by regional sample size) without over-fitting. The search strategy led to a more accurate barcode when compared with traditional SNP (not haplotype)-based approaches, including the incremental addition of SNPs with highest MAF or *Fst*, as well as classification and regression tree^50^ and random forest algorithms^51^ (Supplementary Fig. 7). All statistical analyses were performed using R software (www.r-project.org).

## Author contributions

S.C. and T.G.C. conceived the project. S.A.A., D.F.E., H.O., A.A.-N., L.B.S., D.J.C., S.B., P.M., I.Z., J.-B.O., A.A.D., O.K.D., F.N., A.P., T.B., C.J.D., R.M.F. and C.J.S. contributed to the construction of data. M.D.P., S.C. and T.G.C. analysed the data. C.R. and T.G.C. jointly supervised the research. M.D.P., R.M.F., C.R. and T.G.C. wrote the paper.

## Additional information

**How to cite this article:** Preston, M.D. *et al*. A barcode of organellar genome polymorphisms identifies the geographic origin of *Plasmodium falciparum* strains. *Nat. Commun.* 5:4052 doi: 10.1038/ncomms5052 (2014).

## Supplementary Material

Supplementary Information Supplementary Figures 1-7 and Supplementary Tables 1-5
