# Supplementary material for: A barcode of organellar genome polymorphisms identifies the geographic origin of Plasmodium falciparum strains
Source: Nat Commun. 2014 Jun 13;5:4052. doi: 10.1038/ncomms5052 (PMC4082634; doi:10.1038/ncomms5052)
Supplement: Supplementary Information — Supplementary Figures 1-7 and Supplementary Tables 1-5 [file ncomms5052-s1.pdf]

<u>Supplementary Information for Preston M.D. *et al*.</u>

Supplementary Figure 1 *P. falciparum* organelle sequencing coverage

For each sample, the average mitochondrion (*mt*) and apicoplast *(apico)* genome coverage is compared to the average nuclear (*nuclear*) genome coverage. The *mt* and *apico* coverages are ~22-fold and ~2-fold greater, respectively, than the *nuclear* coverage.

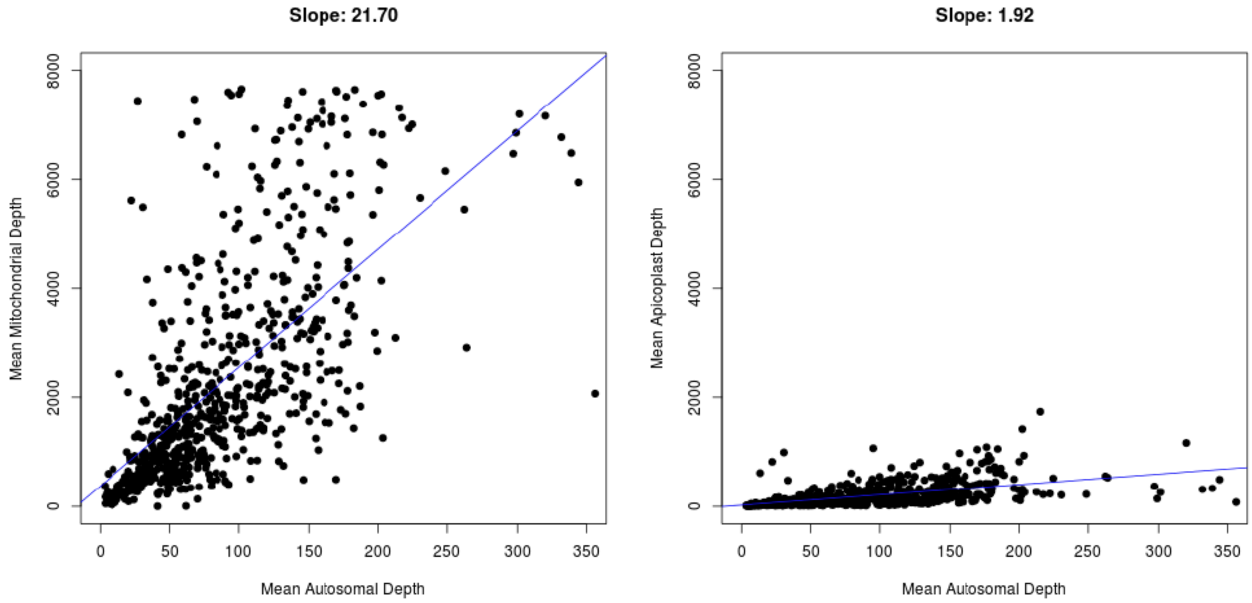

Supplementary Figure 2 Regional genetic differentiation in *P. falciparum*

(a) A linear discriminant analysis using 639 SNPs in 711 *Plasmodium falciparum* samples shows clustering by geographic region: South America, red; West Africa, yellow; East Africa, green; Southeast Asia, blue; and Oceania, purple). There is overlap between the two African regions, and the first two linear discriminant vectors (LD1, LD2) explain 95% of variation. A number of points overlay each other.

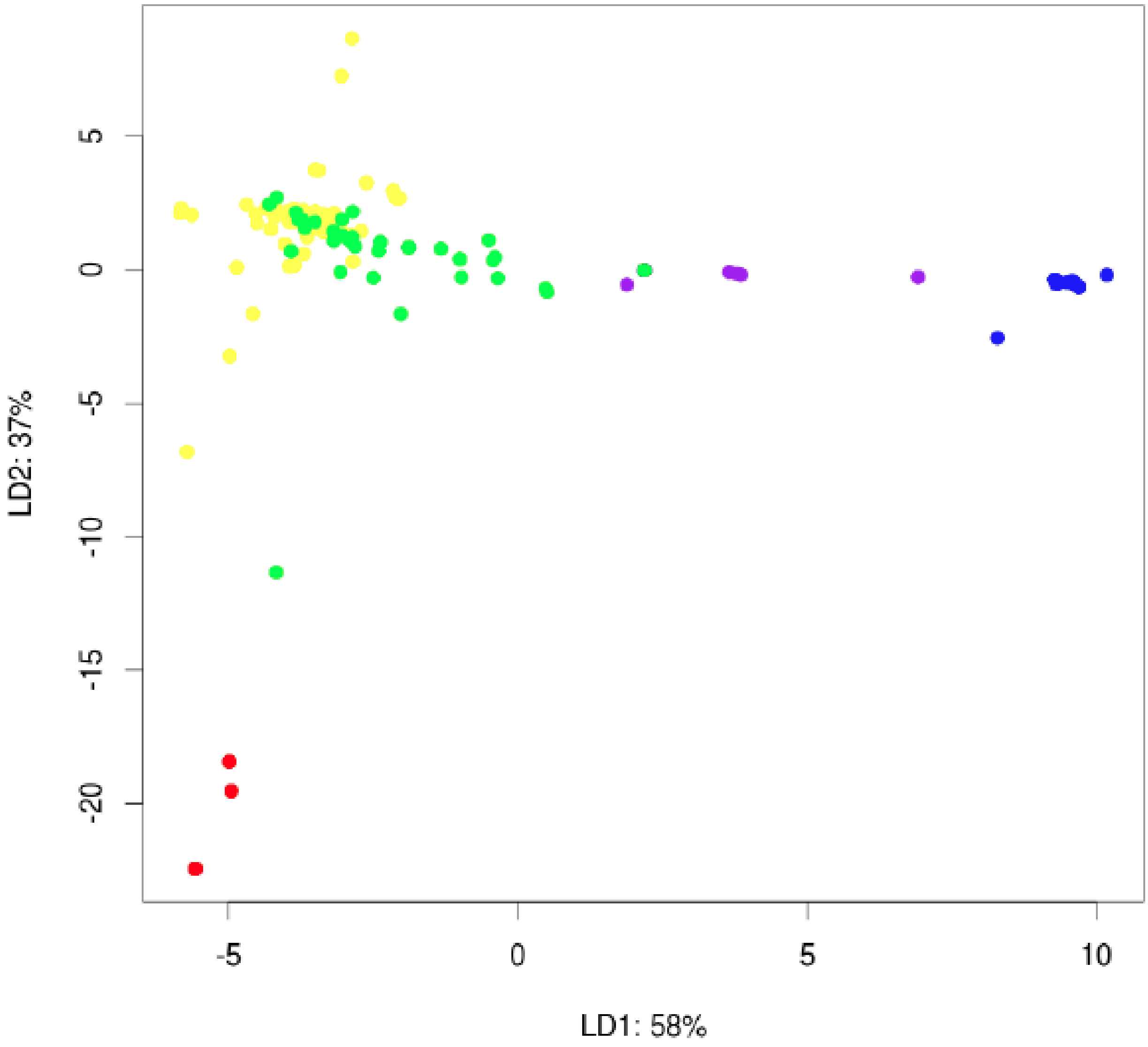

(b) Unrooted phylogenetic tree for 711 *Plasmodium falciparum* samples constructed using 639 SNPs and RaXML (colors as in (a)).

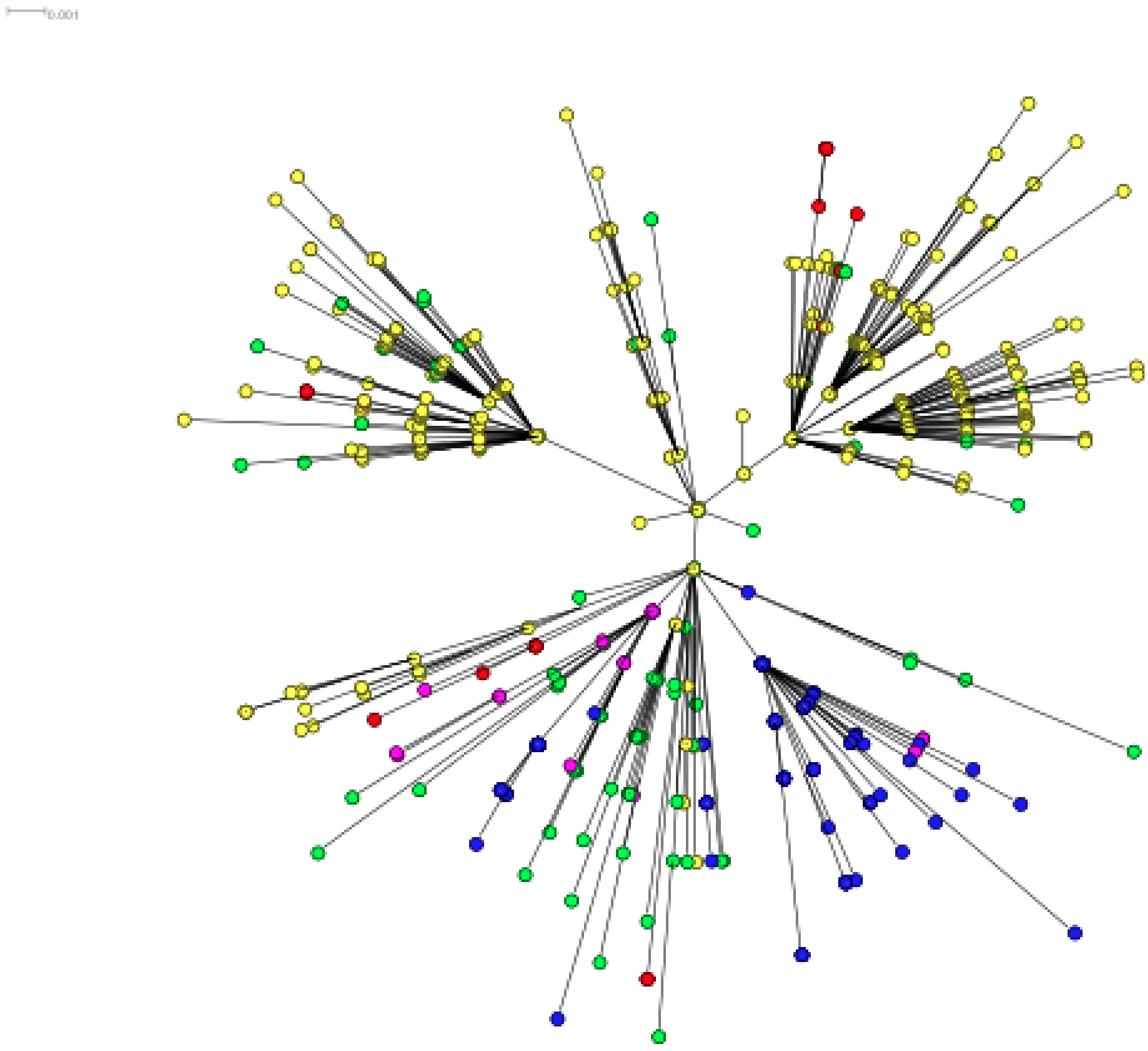

Supplementary Figure 3 Combined minor allele frequency (MAF) and population differentiation *Fst*

Variability at 639 SNPs identified in the organelle genomes of *Plasmodium falciparum*. The combined MAF (>5% labelled, top panel) and *Fst* (>0.1 labelled, bottom panel) values across five geographic regions are shown.

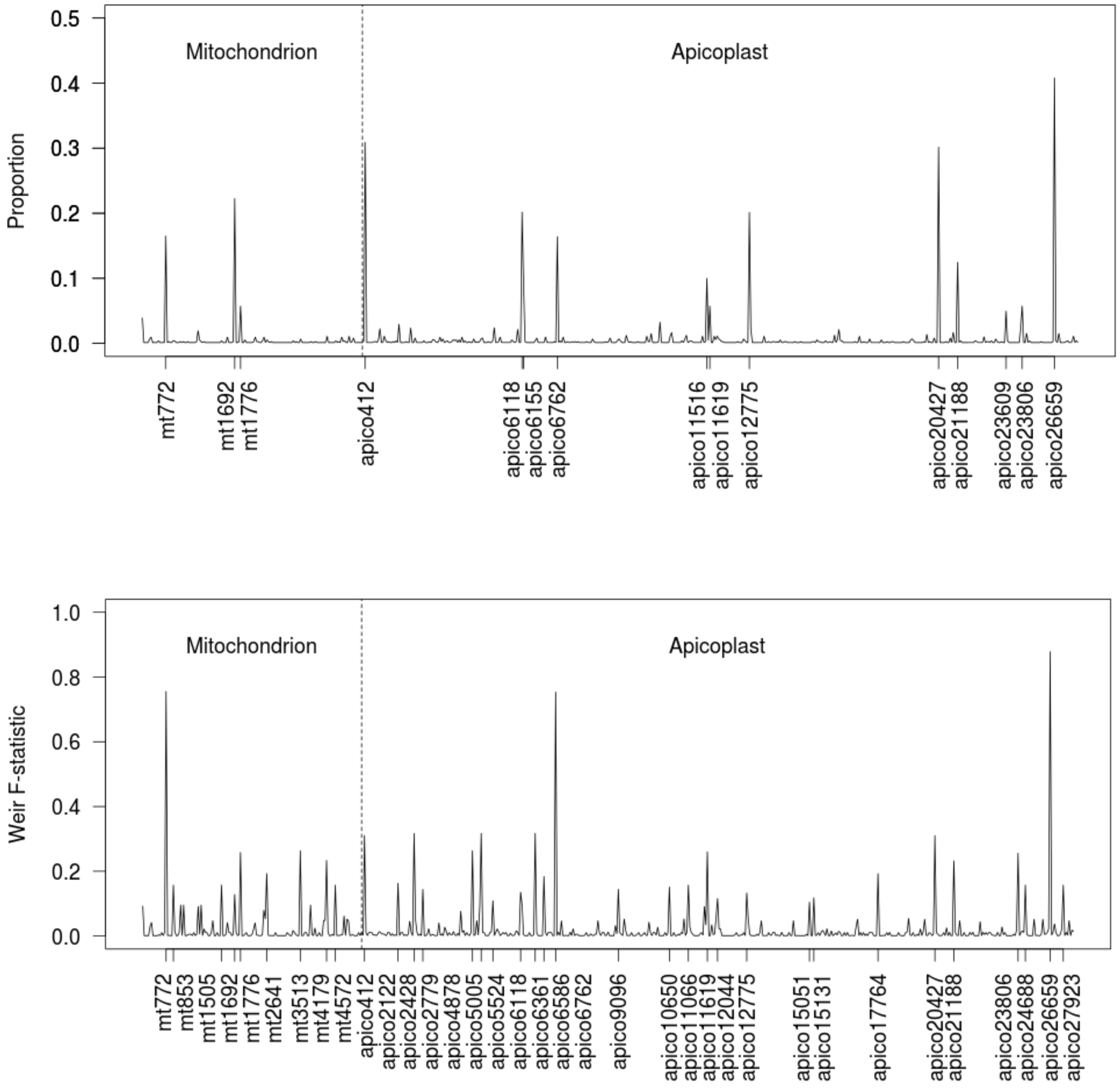

Supplementary Figure 4   Linkage disequilibrium

Pairwise linkage disequilibrium (LD) is presented for all non-rare (minor allele frequency >1% across all samples) and bi-allelic SNPs (2 *mt*, 12 *apico*). The left diagonal represents D’ and the right diagonal represents r^2. The overall mean *D’* value is high (0.972) and similar to mean *D’* values within regions: SAM 1, WAF 0.993, EAF 0.951, SEA 1, and OCE 0.993.

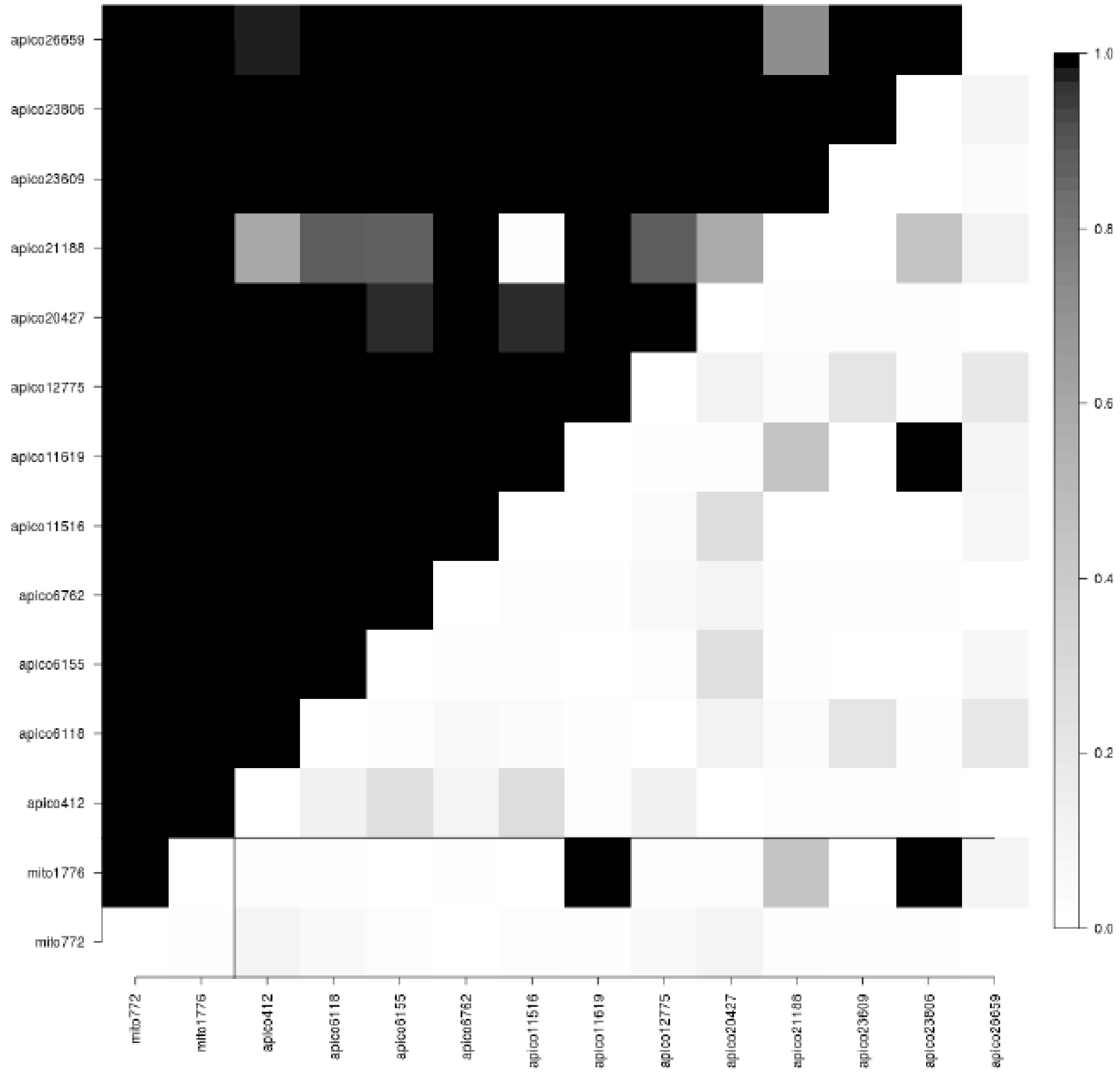

Supplementary Figure 5 **Barcode accuracy**

The accuracy (%) of four sets of haplotypes constructed from mitochondrion (*mt*), apicoplast (*apico*), and nuclear (*nuclear*) genome SNPs *(5)* in five geographic regions. The sets are generated from: (i) 151 *mt* SNPs (146 haplotypes, left bar), (ii) 639 *mt* and *apico* SNPs (290 haplotypes, left middle bar), (iii) 24 *nuclear* SNPs (*5*) (314 compound haplotypes, right middle bar), and (iv) 23 *mt* and *apico* SNPs (34 haplotypes, right bar). The core *mt* haplotype is present in 32.0% of all samples, and is common in four of the five regions: SAM 30.4%, WAF 37.29%, EAF 49.0%, SEA 0%, and OCE 36.0% (left bars, grey). EAF is the most difficult region to assign because 29.6% of samples from EAF share haplotypes with samples from other regions. The 23-SNP barcode has an overall accuracy of 92.1%, and a concordance with the 639-SNP approach of 97.9%.

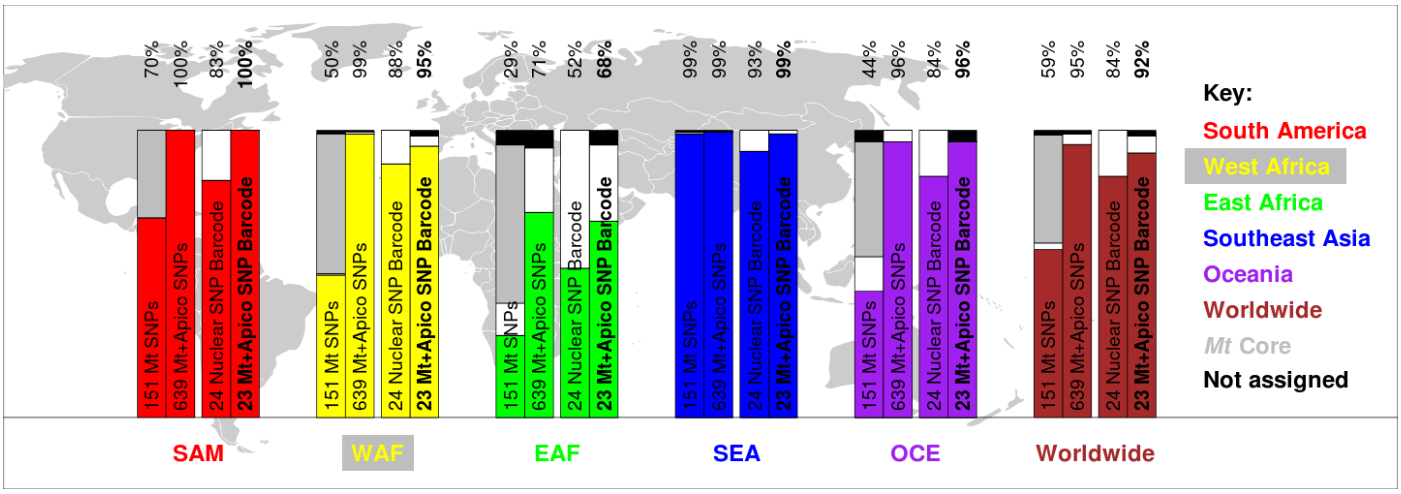

Supplementary Figure 6 *Tajima's D* **measure**

The *Tajima's D* statistic for measuring neutral evolution is calculated using SNP data in genes overall (horizontal lines) and by geographic region (South America, red; West Africa, yellow; East Africa, green; Southeast Asia, blue; Oceania, purple; Overall, black). Negative values indicate more than expected low frequency (rare) alleles, consistent with an expanding population or a purifying selection, as demonstrated strongly in Africa (mean values: *apico* -2.04, *mt* -2.39) and Asia (-1.43, -1.71), and possibly Papua New Guinea (-1.04, -1.37). There is evidence of neutrally mutating population in South America (0.02, 0.19). Overall, there are few positive values (7.9%).

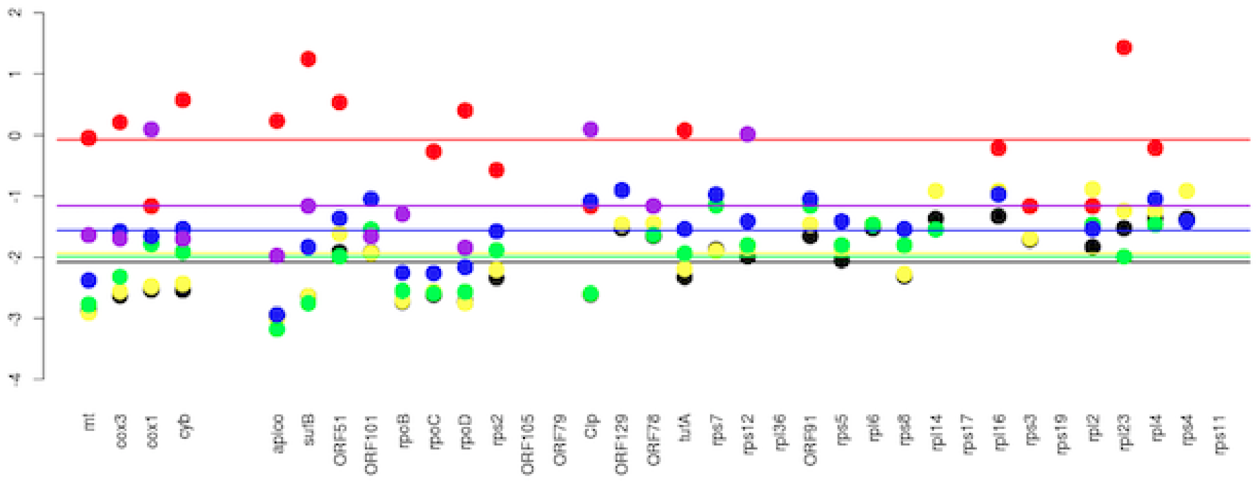

Supplementary Figure 7 **Comparison of alternative barcode SNP Identification strategies**

The accuracy of the iterative SNP haplotype-classification algorithm, weighted (WIBA) and unweighted (IBA) by regional sample size, was compared to the incremental addition of SNPs with high minor allele frequency (MAF) or *Fst*, and classification trees and random forest approaches. Our approach outperforms the alternatives with respect to both accuracy (the percentage of samples correctly identified) or weighted accuracy (assumes all regions of equal size), when considering more than 17 SNPs. The dashed lines reflect the 90% and maximum possible accuracy using all 639 SNPs and 290 haplotypes.

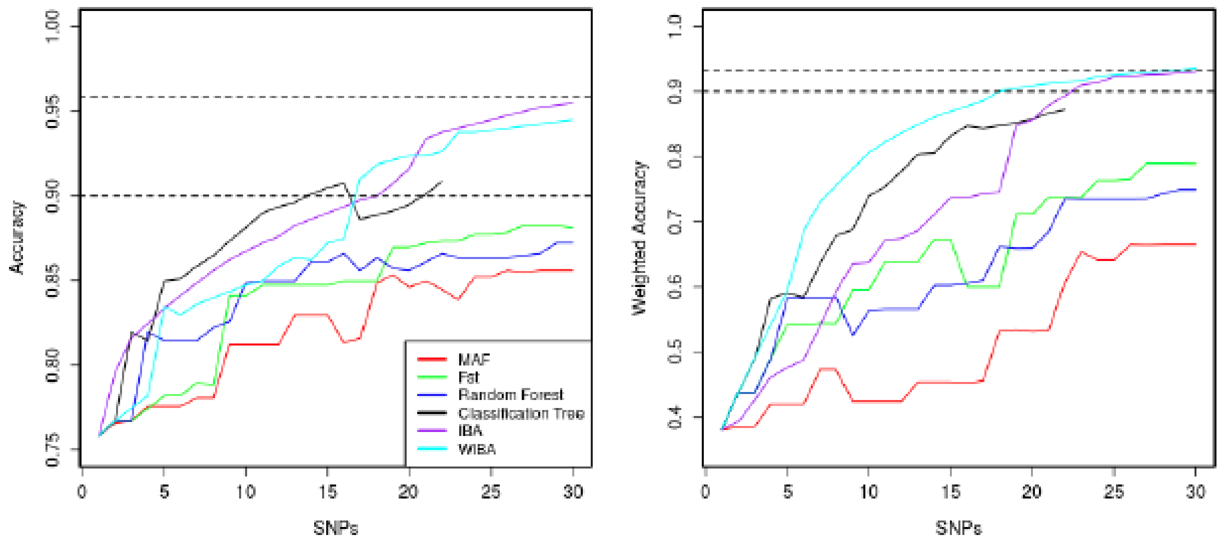

Supplementary Table 1 SNPs detected in *P. falciparum* mitochondrion and apicoplast genomes

The *apico* core is 4.9 times longer than *mt*, but has 3.2 and 2.8 times as many total and region informative (*Fst* >0.1) SNPs, respectively. The ratio of non-synonymous to synonymous SNPs is greater in *apico* (3.5 to 1). Ninety short insertions or deletions (indels) were detected in *apico* (not shown).

Organelle	Length (bp)	Total SNPS	Bi-allelic SNPs	Tri-allelic SNPs	Quad-allelic SNPs	MAF > 0.05	MAF > 0.01	*Fst* > 0.1	Synonymous	Non-synonymous	Genic	RNA	Inter-genic	Haplotypes
mt	5967	151	145	5	1	3	7	9	68	31	99	35	17	146
apico	29430*	488	463	24	1	12	41	25	84	294	378	102	8	271
Total	35397	639	608	29	2	15	48	34	152	325	477	137	25	417

* core genome

Supplementary Table 2 Informative SNPs in *P. falciparum* mitochondrion and apicoplast genomes

The 49 SNPs with minor allele frequency (MAF) >1% in at least one geographic region are listed. Those included in the 23-SNP barcode are bolded.

Organelle	Position	Gene	*P. reichenowi*	3D7 referecne	Alternate	S/NS	Overall MAF	SAM AF	WAF AF	EAF AF	SEA AF	OCE AF	*Fst*
mt	**772**	***cox3***	**C**	**C**	**T**	**N**	**0.166**	**-**	**0.005**	**-**	**0.695**	**0.240**	**0.757**
mt	**853**	***cox3***	**A**	**T**	**G**	**N**	**0.004**	**0.130**	**-**	**-**	**-**	**-**	**0.158**
mt	**973**	***cox3***	**T**	**T**	**A**	**N**	**0.003**	**-**	**-**	**-**	**-**	**0.080**	**0.097**
mt	**1283**	***cox3***	**A**	**A**	**G**	**S**	**0.003**	**-**	**-**	**0.020**	**-**	**-**	**0.022**
mt	1505	-	G	T	G	-	0.004	0.130	-	-	-	-	0.158
mt	1692	-	A	G	ATC	-	0.223	0.261	0.304	0.184	-	0.160	0.129
mt	1776	-	T	T	C	-	0.058	-	-	-	0.250	-	0.259
mt	**2383**	***cox1***	**G**	**G**	**A**	**N**	**0.010**	**-**	**-**	**0.071**	**-**	**-**	**0.081**
mt	2641	*cox1*	T	T	C	S	0.006	-	-	-	-	0.160	0.194
mt	3513	*cyb*	T	T	C	S	0.007	0.217	-	-	-	-	0.265
mt	4179	*cyb*	C	C	T	S	0.011	0.261	0.005	-	-	-	0.235
mt	4572	*cyb*	G	G	A	N	0.004	0.130	-	-	-	-	0.158
apico	412	RNA	A	G	A	-	0.309	0.348	0.466	0.122	-	-	0.311
apico	**2122**	**-**	**A**	**T**	**G**	**-**	**0.030**	**-**	**0.005**	**0.173**	**0.006**	**0.040**	**0.164**
apico	2428	RNA	T	A	G	-	0.009	0.261	-	-	-	-	0.318
apico	2779	RNA	T	G	A	-	0.004	-	-	-	-	0.120	0.145

apico	**4370**	**RNA**	**A**	**T**	**C**	**-**	**0.010**	**-**	**-**	**0.071**	**-**	**-**	**0.078**
apico	**4878**	**RNA**	**T**	**C**	**T**	**-**	**0.007**	**0.217**	**-**	**-**	**-**	**-**	**0.265**
apico	**4945**	**RNA**	**A**	**A**	**G**	**-**	**0.001**	**-**	**-**	**-**	**-**	**0.040**	**0.048**
apico	**5005**	**RNA**	**A**	**A**	**C**	**-**	**0.008**	**0.261**	**-**	**-**	**-**	**-**	**0.318**
apico	5524	*sufB*	A	G	A	N	0.025	0.261	0.027	-	-	-	0.110
apico	**5715**	***sufB***	**T**	**C**	**TA**	**NN**	**0.010**	**-**	**0.005**	**0.031**	**0.006**	**0.040**	**0.013**
apico	6118	*sufB*	T	G	A	N	0.202	0.261	0.289	0.163	-	-	0.136
apico	6155	*sufB*	A	G	A	S	0.093	-	0.157	0.010	-	-	0.095
apico	**6361**	***sufB***	**T**	**C**	**T**	**N**	**0.008**	**0.261**	**-**	**-**	**-**	**-**	**0.318**
apico	6586	*ORF51*	G	G	T	N	0.010	0.217	0.002	-	0.006	-	0.185
apico	6762	*ORF101*	A	G	A	S	0.165	-	-	0.020	0.695	0.240	0.754
apico	**6832**	***ORF101***	**A**	**C**	**TA**	**SS**	**0.010**	**-**	**0.002**	**0.061**	**-**	**-**	**0.048**
apico	**9003**	***rpoB***	**C**	**A**	**G**	**N**	**0.004**	**-**	**-**	**0.031**	**-**	**-**	**0.033**
apico	**9096**	***rpoB***	**A**	**T**	**A**	**N**	**0.004**	**-**	**-**	**-**	**-**	**0.120**	**0.145**
apico	10650	*rpoC*	A	C	T	S	0.017	0.261	0.012	0.010	-	-	0.152
apico	**11066**	***rpoC***	**A**	**G**	**A**	**S**	**0.004**	**0.130**	**-**	**-**	**-**	**-**	**0.158**
apico	11516	*rpoC*	A	C	T	N	0.101	-	0.165	0.031	-	-	0.092
apico	**11619**	***rpoC***	**A**	**T**	**C**	**S**	**0.058**	**-**	**-**	**-**	**0.250**	**-**	**0.261**
apico	**11671**	***rpoC***	**A**	**G**	**AT**	**NN**	**0.011**	**-**	**0.002**	**-**	**0.043**	**-**	**0.035**
apico	12044	*rpoD*	T	C	T	S	0.006	0.130	0.002	-	-	-	0.117
apico	12775	*rpoD*	A	T	C	N	0.202	0.261	0.287	0.173	-	-	0.134
apico	15051	*rps2*	T	A	T	N	0.003	0.087	-	-	-	-	0.105
apico	**15131**	***rps2***	**T**	**C**	**T**	**N**	**0.006**	**0.130**	**-**	**0.010**	**-**	**-**	**0.119**
apico	17764	*Clp*	T	T	C	S	0.006	-	-	-	-	0.160	0.194

apico	20427	*tufA*	A	A	G	N	0.302	0.348	0.459	0.112	-	-	0.311
apico	**20831**	***rps7***	**T**	**G**	**A**	**N**	**0.008**	**-**	**0.005**	**0.041**	**-**	**-**	**0.026**
apico	**21188**	***rps12***	**A**	**G**	**A**	**N**	**0.125**	**-**	**0.035**	**0.143**	**0.256**	**0.680**	**0.233**
apico	23609	*rps8*	A	T	C	N	0.050	-	0.070	0.071	-	-	0.029
apico	**23803**	***rps8***	**A**	**T**	**C**	**S**	**0.024**	**-**	**0.040**	**0.010**	**-**	**-**	**0.019**
apico	23806	*rps8*	A	T	C	S	0.058	-	-	-	0.250	-	0.257
apico	24688	*rpl16*	C	T	A	N	0.004	0.130	-	-	-	-	0.158
apico	**26659**	***rpl23***	**C**	**A**	**G**	**N**	**0.408**	**0.391**	**0.087**	**0.694**	**-**	**-**	**0.879**
apico	27923	RNA	G	A	T	-	0.004	0.130	-	-	-	-	0.158

Supplementary Table 3 Regional genetic variation in *P. falciparum* mitochondrion and apicoplast genomes

Sample number, SNP, and haplotype distributions are stratified by geographical region: SAM, South America; WAF, West Africa; EAF, East Africa; SEA, Southeast Asia; and OCE, Oceania. There are more private SNPs in WAF, fewer private haplotypes in EAF and OCE, and greatest genotypic richness and genetic diversity in EAF and SAM.

Region	No. Samples	No. SNPs	No. Private SNPs	Private SNPs (%)	No. Haplotypes	No. Private haplotypes	Private haplotype (%)	Genotypic richness*	Diversity (π)**
SAM	23	35	18	51.4	13	**13**	100	0.545	0.000210
WAF	401	419	331	79.0	190	184	96.8	0.472	0.000043
EAF	98	186	115	61.8	51	44	86.3	0.515	0.000120
SEA	164	89	63	70.8	33	32	97.0	0.196	0.000051
OCE	25	30	16	53.3	11	9	81.8	0.417	0.000107
Overall	711	639	543	85.0	290	282	97.2	0.407	0.000106

* (no. haplotypes -1)/ (no. samples -1)

** SAM (*apico* 0.000126, *mt* 0.000621), WAF (*apico* 0.000027, *mt* 0.000120), EAF (*apico* 0.000080, *mt* 0.000316), SEA (*apico* 0.000036, *mt* 0.000120), OCE *(apico* 0.000046, *mt* 0.000407)

Supplementary Table 4 **Barcode accuracy in identifying the origin of *P. falciparum* samples**

The predicted geographic origins (SAM, South America; WAF, West Africa; EAF, East Africa; SEA, Southeast Asia; OCE, Oceania) of *P. falciparum* samples using the 23-SNP barcode. The overall accuracy is 92.1% (655/711). Unique assignment was not possible for 14 samples. Only three samples outside Africa were incorrectly assigned, and 7.9% (38/499) of African samples were incorrectly assigned to WAF and EAF.

Region	SAM	WAF	EAF	SEA	OCE
SAM	**23**	0	0	0	0
WAF	0	**379**	24	0	0
EAF	0	14	**67**	1	0
SEA	0	0	0	**162**	0
OCE	0	0	2	1	**24**
Not assigned	0	8	5	0	1
Actual	23	401	98	164	25
Accuracy %	100	94.5	68.4	98.8	96.0

Supplementary Table 5 Evidence of purifying or positive selection using the Ka/Ks measure

Organelle	Gene	No. SNPs	Gene length	*Ka/Ks*
mt	*cox3*	39	753	0.291
mt	*cyb*	29	1131	0.148
mt	*cox1*	31	1437	0.067
apico	***rps8***	**17**	**387**	**7.296**
apico	***rps7***	**8**	**429**	**5.866**
apico	***tufA***	**18**	**1233**	**4.880**
apico	*rps2*	19	684	3.235
apico	*rpoB*	64	3075	3.153
apico	*rps12*	9	369	2.880
apico	*rpl2*	7	738	2.443
apico	*Clp*	37	2301	2.046
apico	*sufB*	39	1413	1.988
apico	*rpoC*	37	1728	1.425
apico	*rps5*	10	720	1.333
apico	*ORF101*	8	306	1.131
apico	*ORF129*	4	390	0*
apico	*rpl6*	4	507	0*
apico	*rpl14*	3	345	0*
apico	*ORF78*	5	237	Inf**
apico	*rpl23*	4	228	Inf**
apico	*rpl4*	3	573	Inf**

apico	*rpl16*	3	390	Inf**
apico	*rps3*	6	645	Inf**
apico	*ORF51*	8	156	Inf**

Ka/Ks is the ratio of the number of non-synonymous (NS) substitutions per NS site (*Ka*) to the number of synonymous (S) substitutions per synonymous site (*Ks*); *Ka/Ks* >> 1 implies positive selection, *Ka/Ks* << 1 implies purifying selection, * no S mutations, ** no NS mutations
